# Microstructure and Mechanical Properties of Laser-Welded DP Steels Used in the Automotive Industry

**DOI:** 10.3390/ma14020456

**Published:** 2021-01-19

**Authors:** Hanbing He, Farnoosh Forouzan, Joerg Volpp, Stephanie M. Robertson, Esa Vuorinen

**Affiliations:** 1 Division of Material Science, Luleå University of Technology, 971 87 Luleå, Sweden; farnoosh.forouzan@ltu.se (F.F.); Esa.Vuorinen@ltu.se (E.V.); 2 Division of Product and Production Development, Luleå University of Technology, 971 87 Luleå, Sweden; jorg.volpp@ltu.se (J.V.); stephanie.robertson@ltu.se (S.M.R.)

**Keywords:** laser welding, dual phase steel, similar/dissimilar welded joints, microhardness, tensile properties, fatigue

## Abstract

The aim of this work was to investigate the microstructure and the mechanical properties of laser-welded joints combined of Dual Phase DP800 and DP1000 high strength thin steel sheets. Microstructural and hardness measurements as well as tensile and fatigue tests have been carried out. The welded joints (WJ) comprised of similar/dissimilar steels with similar/dissimilar thickness were consisted of different zones and exhibited similar microstructural characteristics. The trend of microhardness for all WJs was consistent, characterized by the highest value at hardening zone (HZ) and lowest at softening zone (SZ). The degree of softening was 20 and 8% for the DP1000 and DP800 WJ, respectively, and the size of SZ was wider in the WJ combinations of DP1000 than DP800. The tensile test fractures were located at the base material (BM) for all DP800 weldments, while the fractures occurred at the fusion zone (FZ) for the weldments with DP1000 and those with dissimilar sheet thicknesses. The DP800-DP1000 weldment presented similar yield strength (YS, 747 MPa) and ultimate tensile strength (UTS, 858 MPa) values but lower elongation (EI, 5.1%) in comparison with the DP800-DP800 weldment (YS 701 MPa, UTS 868 MPa, EI 7.9%), which showed similar strength properties as the BM of DP800. However, the EI of DP1000-DP1000 weldment was 1.9%, much lower in comparison with the BM of DP1000. The DP800-DP1000 weldment with dissimilar thicknesses showed the highest YS (955 MPa) and UTS (1075 MPa) values compared with the other weldments, but with the lowest EI (1.2%). The fatigue fractures occurred at the WJ for all types of weldments. The DP800-DP800 weldment had the highest fatigue limit (348 MPa) and DP800-DP1000 with dissimilar thicknesses had the lowest fatigue limit (<200 MPa). The fatigue crack initiated from the weld surface.

## 1. Introduction

Advanced high strength steel (AHSS) is the fastest growing material group in today’s automotive industry, because of its high strength to weight ratio performance, which allows the car makers to produce thinner components and, thereby, reduce the fuel consumption [1,2,3]. Dual phase (DP) steel group is among the AHSS family widely used in the crash zones of the vehicle due to its high energy absorption ability [3,4,5]. During the past two decades, as the most essential joining technique used in the automotive industry, laser welding has become popular because of its ability to increase the production rate and allow great flexibility of the joint design, without increasing the metallurgical heterogeneities across the weldments [6,7].

The interest has increased on the welding of dissimilar materials with the purpose to reduce both production and operation costs and optimize the properties, such as strength and hardness. Considering the advantages of the laser welding, such as the small heat-affected zone, low heat input, small distortion level etc. [6,7], it is attracting more attentions due to its potential to achieve this purpose. However, during the welding process, element mixing occurs and new phases can be formed. The appearance of the new phases produces the differences between the base material (BM) and the joint, which can significantly affect the overall properties of the welded material, e.g., poor mechanical strength and fatigue [8,9]. Additional challenges may occur when welding the dissimilar materials because of the different physical and chemical properties of the BMs, such as thermal expansion coefficients, melting points, and mechanical properties, as well as the formation of intermetallic compounds [10,11,12]. The thermal cycles of welding processes cause changes in the microstructures of DP steels, leading to the formation of a fusion zone (FZ) and a heat-affected zone (HAZ) in the welded joint (WJ). Due to the high heating and cooling rates, the FZ is dominantly composed of martensite [13]. The formation of the FZ leads to significant increase in hardness. However, a soft zone (SZ) far from the fusion line at the HAZ was observed. Since the appearance of this SZ could have an adverse effect on mechanical properties, extensive studies have been conducted to investigate this phenomenon [13,14,15,16,17]. Two temperature softening mechanism dependences have been reported [13,14,15]. At tempering temperature, martensite will be tempered, and precipitation of carbides can occur resulting in the softening, while at the inter-critical temperature, the softening is caused by the decrease in the final martensite amount due to the transformation of martensite into austenite and subsequent possible formation of ferrite, bainite or retained austenite microconstituents during cooling [13,14,15,16,17]. The extent of softening depends on the heat input as well as the grade of steel [16,17,18]. More severe softening of the DP980-WJ than of the DP600-WJ was observed due to the higher amount of martensite in the former [17].

Heterogeneous microstructures and mechanical properties may greatly change the overall properties of the welded components. Compared to the BM, the ultimate tensile strength (UTS) was reported to be decreased or almost unchanged, whereas an increase in the yield strength (YS) has been reported [8,16,18]. Simulation results revealed that UTS of the joint decreases with an increase in HAZ width and the softening degree [15]. Significant reduction in elongation was widely observed [8,9]. Wang et al. [15] observed an apparent extension of the HAZ, not in BM and FZ, by comparing their specific length before and after tensile tests, thereby indicating that the significant reduction of ductility was a result from the inharmonious deformation in the different zones. The tensile fracture location was reported to occur at the BM and at the SZ in HAZ [8,9,16,19]. The fracture location is more likely to appear at the BM, with narrower HAZ width, lower magnitude of softening as well as with wider FZ width [15,18,19,20]. The different results reported were probably due to the different welding parameters, as observed that the weld width increases with increased heat input [18,19]. The fatigue property of DP WJs is attracting more attention [21,22,23,24,25,26]. The geometry of WJ play an important role on the fatigue strength. Furthermore, the fatigue facture appeared at the SZ at higher stress amplitude, whereas it rarely occurred at the SZ at lower amplitude.

Laser welding of DP steels is well documented in the literature and are mostly focused on steels with similar base metals and thicknesses as well as similar tensile properties. It is still a challenge when welding the joint in combination with dissimilar material/thickness. Therefore, it is necessary to analyze the welded material to understand this technology. Since the WJ can affect the final mechanical properties, it is important to analyze the WJ as well as its effect on the mechanical phenomena, especially the dissimilar combination of material and thickness. In this regard, this study was aimed at characterizing the fiber laser welded DP steel joints in similar/dissimilar combinations of materials and thicknesses to understand the effect of WJ on the static and dynamic performance of weldments. Furthermore, differences between microstructure and mechanical properties of these weldments have been compared.

## 2. Materials and Methods

### 2.1. Materials and Specimen Fabrication

The materials used in this study are cold rolled square profile tubes (edge length 40 mm) of DP800 and DP1000 steels, with a thickness of 1.3 and 2.1 mm. The chemical compositions and mechanical properties of the base materials DP800 and DP1000 are presented in Table 1. The WJs were fabricated from similar BM with similar/dissimilar thicknesses or in combination of dissimilar BMs with similar/dissimilar thicknesses. Details of the different WJs performed are shown in Table 2.

**Figure 1:**
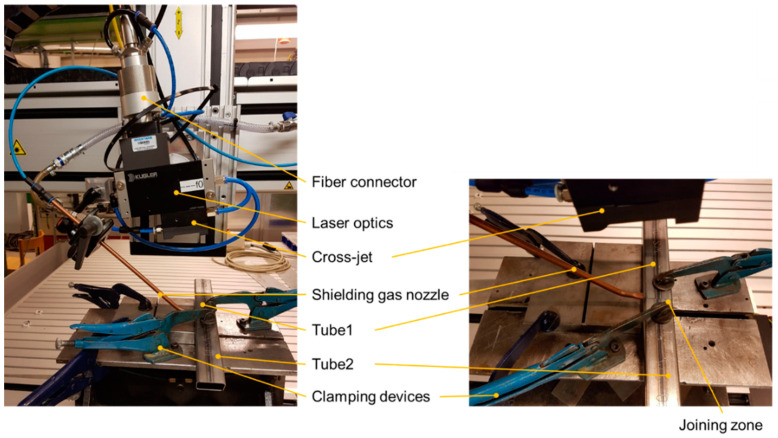
Setup for the laser welding experiments of tubes showing the large view with all components (**left**) and the detailed view of the clamped work piece (**right**).

Three types of tubes were tested, differing in type and wall thickness. The butt-welded joints were produced using a single-pass single-beam laser weld using an IPG fiber laser (YRL-15000, cw, IPG Laser GmbH, Carl-Benz- Straße 28, 57299 Burbach, Germany) in combination with a Precitec YW52 optics (by Precitec GmbH & Co. KG, Draisstraße 1, 76571 Gaggenau, Germany), with the welding parameters presented in Table 3. The line energy is 60 J/mm. The setup of the laser welding experiments used are shown in Figure 1. Laser welding was performed in the focal position with a laser beam angel of 7° applying argon (purity 99.998%) as shielding gas at 18 L/min to cover the processing zone from the surrounding air. The square tubes to be welded were clamped in a “zero-” gap configuration. No additional edge preparation was used to enhance fitting. One weld seam was produced for each parameter set. The material was cleaned using ethanol before welding to avoid impacts of contamination.

**Figure 2:**
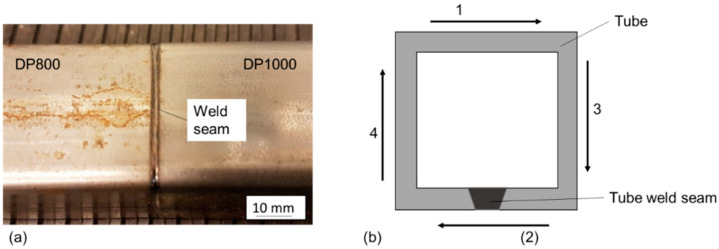
(**a**) Top view of a laser-welded joint on tubes with dissimilar materials; (**b**) Welding sequence visualization.

A typical WJ on a tube is shown in Figure 2a. For mechanical testing, the sides 1, 3 and 4 were used due to the tube weld seam alongside 2 (Figure 2b). For minimization of distortion effects on subsequent welding, the welding sequence in Figure 2b was chosen.

Visual testing was conducted in order to identify visible defects such as surface cracks or lack of fusion. However, the produced weld seams showed sound appearances.

### 2.2. Microstructure Investigation and Mechanical Testing

The specimens used in the microstructure and microhardness investigations were grounded, polished and etched in 3% Nital solution to reveal the microstructures. Microstructure investigation was performed using optical microscopy (OM) Nikon Eclipse MA200 (Nikon Instech Co., Ltd., Tokyo, Japan, Japan). Vickers microhardness test was performed at load of a 500 g and a dwell time of 15 s across the base metal, HAZ, and weld metal. The fatigue fracture surfaces were examined with a scanning electron microscopy (SEM) JEOL JSM-IT300LV (JEOL Ltd. Tokyo, Japan).

**Figure 3:**
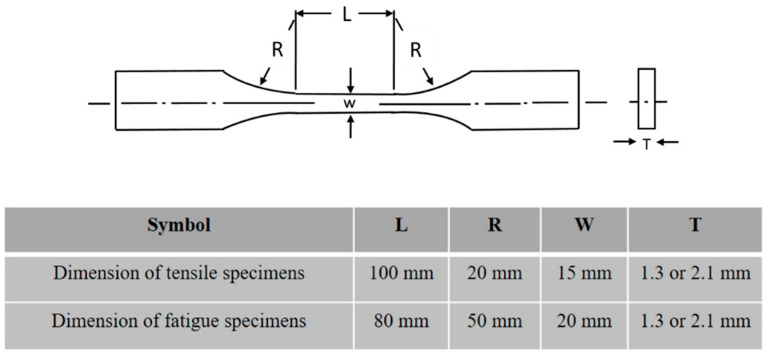
Dimensions of test specimens for tensile- and fatigue-tests, with welds in the center of L-gauge.

Tensile and fatigue test samples were prepared following the ASTM E466-96 standard [27] with the geometry and dimensions shown in Figure 3. The samples were sectioned from the welded work pieces with the weld positioned at the center of the gauge length. The fatigue tests were carried out using Instron servo-hydraulic universal testing machine (Instron. Co. UK., High Wycombe, UK) equipped with data acquisition and hydraulic pressure grips at a frequency of 20 Hz and with a stress ratio of 0.1.

## 3. Results

### 3.1. Appearance of Welded Joints and Microhardness Results

**Figure 4:**
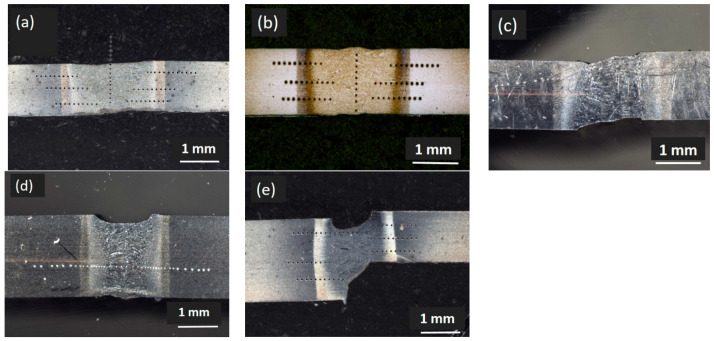
Crosssections of the different types of WJs. (**a**) DP800/1.3-DP800/1.3; (**b**) DP1000/1.3-DP1000/1.3; (**c**) DP800/1.3-DP1000/1.3; (**d**) DP800/2.1-DP800/2.1; (**e**) DP800/2.1-DP1000/1.3.

The cross-sections of the different types of WJs are displayed in Figure 4. The results showed that, under the same laser welding conditions, the weld center of DP1000/1.3-DP1000/1.3 and DP800/1.3-DP1000/1.3 were convex, while concave weld center was observed in the DP800/2.1-DP800/2.1 and DP800/2.1-DP1000/1.3. These weld centers were different from that of DP800/1.3-DP800/1.3, for which a flat weld center was observed. The different weld seam appearances may result from slight alignment differences due to the clamping in technical and not nominal zero-gap configuration. A slight gap can lead to a concave seam appearance.

**Figure 5:**
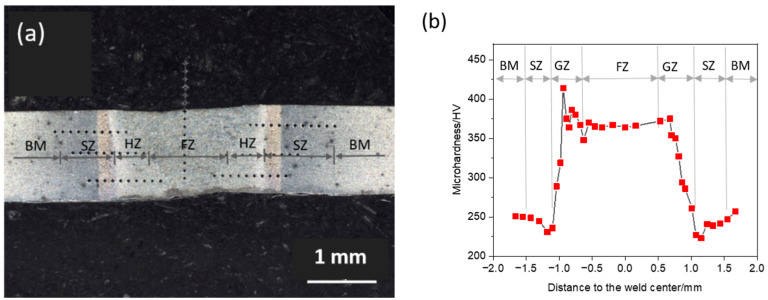
(**a**) Different zones in DP800/1.3-DP800/1.3 WJ, and (**b**) microhardness across the different zones.

A typical DP800/1.3-DP800/1.3WJ was selected to demonstrate the different zones in the WJ, since similar characteristics were observed among the different types of WJs, namely fusion zone (FZ), hardening zone (HZ) and softening zone (SZ), as illustrated in Figure 5a. The microhardness along the different zones were measured, as shown in Figure 5b. The hardness in the FZ displayed an approximate value of 370 HV, while the highest hardness appeared at the boundary between the FZ and HZ. A hardness drop was found in the HZ, the minimum value around 220 HV appeared in the SZ. A hardness increase was observed from the SZ to the BM region.

**Figure 6:**
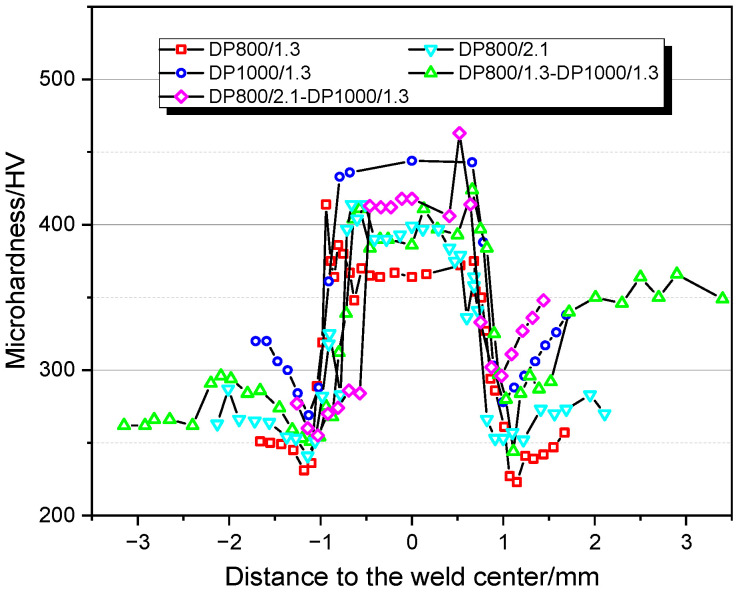
Measurement points of microhardness at the welded joints across the BM, SZ, HZ and FZ. BM: base metal, SZ: softening zone, HZ: hardening zone, and FZ: fusion zone.

The microhardness profiles across the five types of WJs are shown in Figure 6. Consistent trends were found for the different types of WJs, featuring peaks in the HZ and valleys in the SZ. The lowest microhardness values in DP800/1.3-DP800/1.3 and DP1000/1.3-DP1000/1.3 WJs were between 220 and 275 HV, which was 8 and 20% lower in comparison with the corresponding base material. In addition, the width of SZ in DP800/1.3-DP800/1.3 WJ was around 0.49 mm, narrower compared with the DP1000/1.3-DP1000/1.3 WJ with the value of ~0.75 mm. This agrees with the results reported that the softening degree and the size of soften zone was increased with increasing strength grade of the steels [17,18]. The DP800/2.1-DP800/2.1 WJ had wider SZ (~0.54 mm) and higher microhardness in the FZ compared with DP800/1.3-DP800/1.3 WJ. A comparison of the WJs for combinations with dissimilar materials with those of similar materials showed that the SZ of DP800/1.3-DP1000/1.3 WJ at DP800 side was wider, while similar width was observed at the DP1000 side.

### 3.2. Microstructures

**Figure 7:**
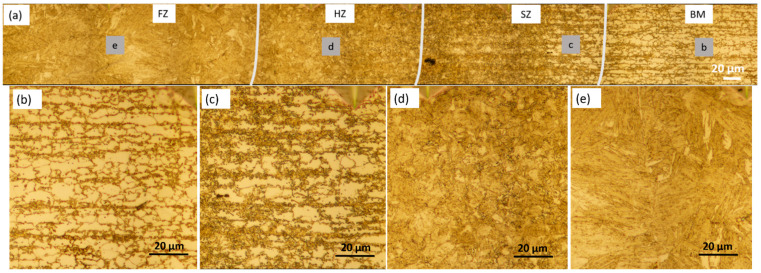
Optical microscope images of WJ (**a**), BM (**b**), SZ (**c**), HZ (**d**) and FZ (**e**) in the DP800/1.3-DP800/1.3 WJ.

**Figure 8:**
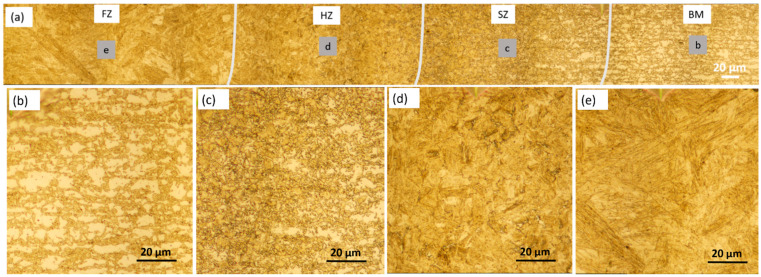
Optical microscope images of WJ (**a**), BM (**b**), SZ (**c**), HZ (**d**) and FZ (**e**) in the DP1000/1.3-DP1000/1.3 WJ.

Figure 7 and Figure 8 present optical micrographs taken at typical locations in the WJs. As shown in the figures, different zones in the weld metals can be distinguished. For each specific zone, all WJs exhibited similar microstructural constituents and morphologies. Therefore, a representative DP800/1.3-DP800/1.3 WJ and a DP1000/1.3-DP1000/1.3 WJ were selected to illustrate the microstructures of the different zones in the WJs.

The BM DP800 was characterized by martensitic islands in a ferrite matrix, as shown in Figure 7b. The microstructures at the SZ were composed of coarse grains of ferrite and probably tempered martensite as shown in Figure 7c, with minimum hardness values in the hardness profiles. The volume fraction of ferrite towards the FZ was decreased, indicating partial transformation of ferrite. Martensite and ferrite at the HZ were observed, and the grain size was bigger near the FZ (Figure 7d). The FZ was dominated by lath martensite, and the grain size was larger compared to that in BM (Figure 7e).

Similar to DP800/1.3-DP800/1.3 WJ, different zones can be distinguished in DP1000/1.3-DP1000/1.3 WJ and similar microstructure characteristics at specific zones were found, as shown in Figure 8. The observed volume fraction of martensite was higher in the BM of DP1000 compared with DP800 (Figure 8b). A lower volume fraction of ferrite was also observed in the SZ in comparison with BM (Figure 8c). The HZ was dominated by martensite with varying grain size (Figure 8d) and lath martensite was found in the FZ (Figure 8e).

### 3.3. Tensile Test Results

**Figure 9:**
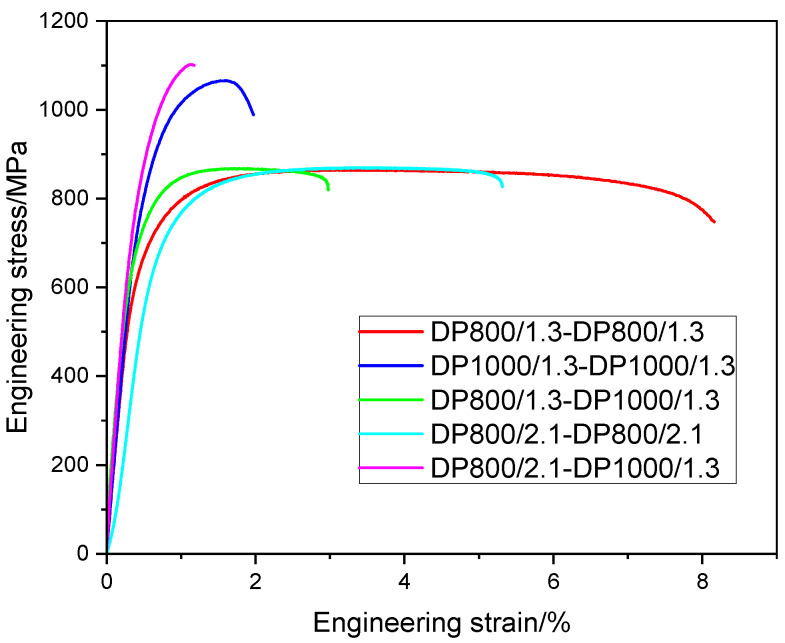
Stress-strain curves of the tensile tested weldments.

The representative engineering stress versus engineering strain plots of the tensile tested weldments are displayed in Figure 9. The stress–strain curve of the DP800/1.3-DP1000/1.3 weldment was located between that of DP800/1.3-DP800/1.3 and DP1000/1.3-DP1000/1.3 weldments.

The average tensile test results are listed in Table 4. DP800/1.3-DP800/1.3 weldment exhibited 701 MPa, 868 MPa, and 7.9% for yield strength (YS), ultimate tensile strength (UTS) and elongation at fracture (EI) respectively, which is close to the values of the BM DP800 (YS 686 MPa, UTS 815 MPa, and EI 7.9%). DP800/2.1-DP800/2.1 weldment showed the values of 742 MPa (YS) and 861 MPa (UTS), almost similar to that of BM DP800 and DP800/1.3-DP800/1.3 weldment. Meanwhile, a lower elongation at fracture was observed. For DP1000/1.3-DP1000/1.3 weldment, the YS was 883 MPa, 16% higher than that of the BM, and the UTS was 1034 MPa, similar to the BM DP1000, while a high reduction of EI was observed.

The YS of DP800/1.3-DP1000/1.3 weldment was slightly increased compared with BM DP800 and DP800/1.3-DP800/1.3 weldment, but lower than BM DP1000 and DP1000/1.3-DP1000/1.3 weldment. The value of UTS for DP800/1.3-DP1000/1.3 weldment was close to that of DP800/1.3-DP800/1.3 weldment, but lower compared with DP1000/1.3-DP1000/1.3 weldment. In addition, the EI of DP800/1.3-DP1000/1.3 weldment was between that of DP800/1.3-DP800/1.3 and DP1000/1.3-DP1000/1.3 weldments. DP800/2.1-DP1000/1.3 weldment had the highest YS and UTS (955, 1075 MPa), but lowest elongation (1.2%), in comparison with other weldments. The value of elongation of the DP800/2.1-DP1000/1.3 weldment indicates that the weldment become brittle and it collapses almost without plastic deformation.

It should be noted that the fracture was located at the BM for DP800/1.3-DP800/1.3, DP800/2.1-DP800/2.1 and DP800/1.3-DP1000/1.3 weldments, while it occurred at the WJ for DP1000/1.3-DP1000/1.3 and DP800/2.1-DP1000/1.3 weldments.

### 3.4. Fatigue Test Results

**Figure 10:**
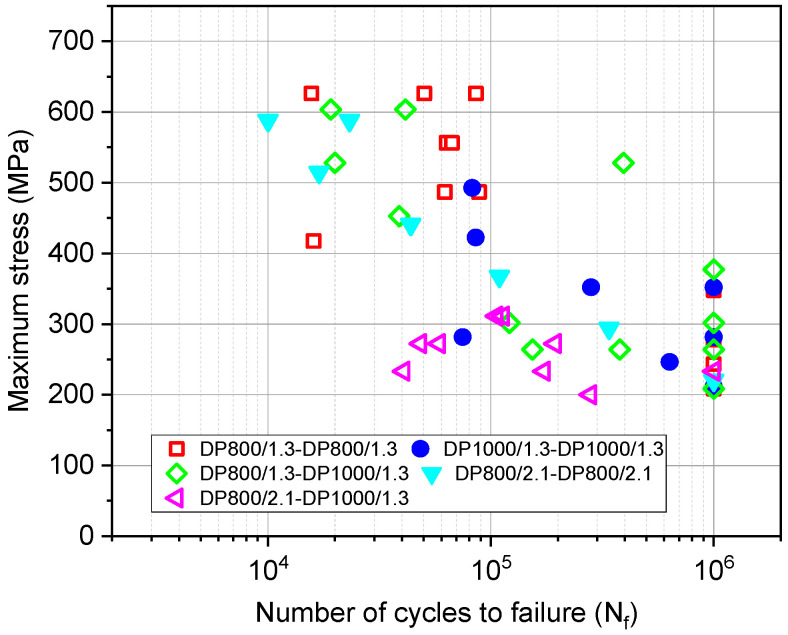
Fatigue test results of the welded DP800/1.3, DP800/2.1, DP1000/1.3, DP1000/1.3-DP800/1.3 and DP800/2.1-DP1000/1.3 samples, tested at R = 0.1, 20 Hz, and RT.

Fatigue test results of the weldments obtained at R = 0.1, 20 Hz, and room temperature (RT) are plotted in Figure 10. The limit for run out (RO) was set to 1 million cycles (10^6^). The stress level target for the RO was set to 200 MPa. The results show that, although the points were scattered, probably due to the defects introduced during the welding process, it still can be observed that the DP800/2.1-DP800/2.1 weldment had a lower fatigue life than the DP800/1.3-DP800/1.3 weldment at both high and low levels of stress. An interesting finding in the comparison of these two weldments is that the thicker sheet combination (2.1 mm) shows lower scatter of the results in comparison with the thinner sheet (1.3 mm). The DP800/2.1-DP1000/1.3 weldments exhibited the lowest fatigue life in comparison with the other weldments with a RO stress below 200 MPa.

The fatigue limits (ROs) are tabulated in Table 4. It is shown that the fatigue limit of the DP800/1.3-DP800/1.3 weldment was 348 MPa, which was significantly higher than that of the DP1000/1.3-DP1000/1.3 and DP800/1.3-DP1000/1.3 weldments with the value of approximate 210 MPa. Even though the same material was used, the fatigue limit of DP800/2.1-DP800/2.1 weldment was obtained to be 221 MPa, lower than DP800/1.3-DP800/1.3 weldment.

**Figure 11:**
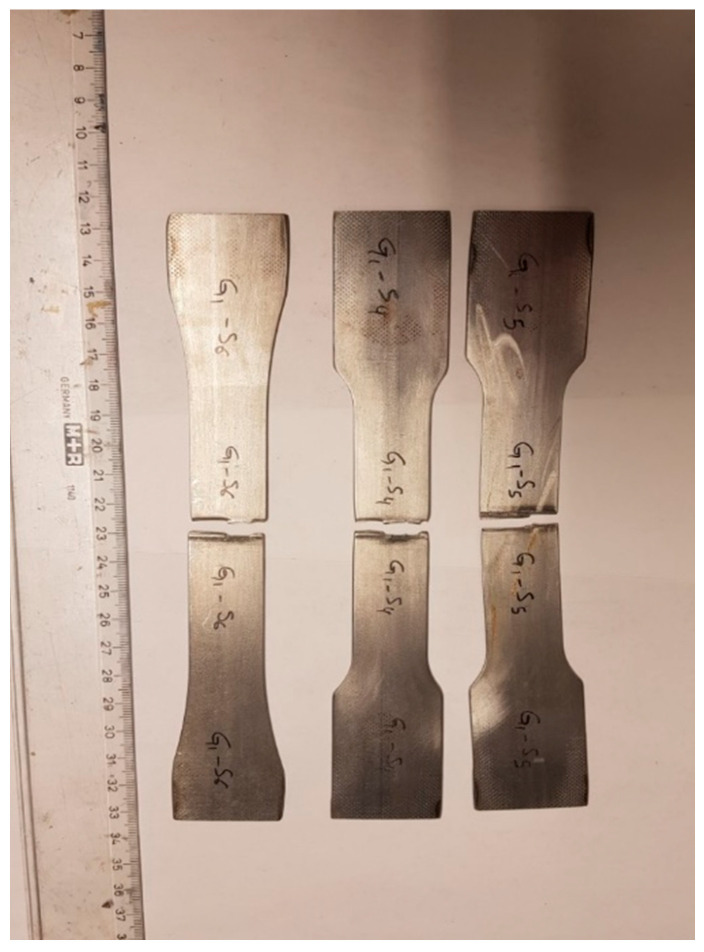
Typical fatigue failure locations.

The typical fatigue failure locations are shown in Figure 11. It was observed that the fracture occurred at the WJ. All types of weldments presented the same behavior.

### 3.5. Fractography

**Figure 12:**
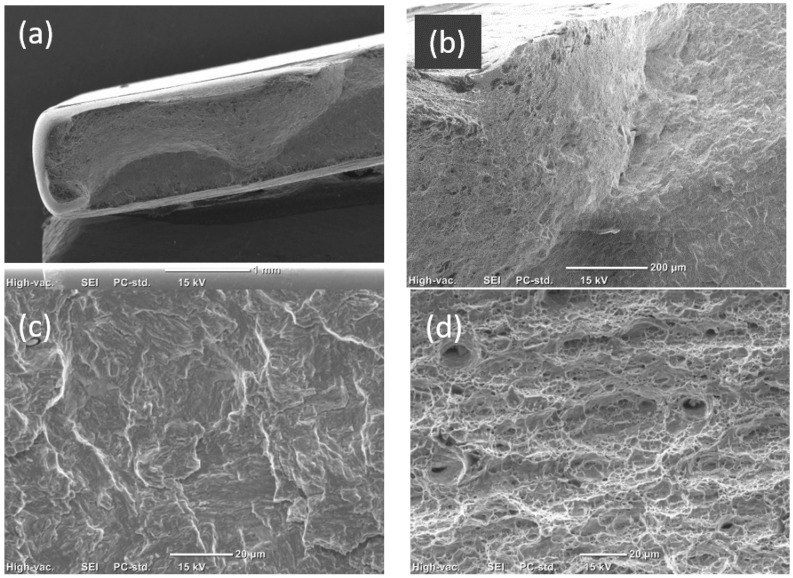
SEM images of the fatigue fracture surface of a DP800/1.3-DP800/1.3 weldment under the maximum stress of 626 MPa. (**a**) Overall morphology of fatigue fracture, (**b**) crack initiation region, (**c**) crack propagation region, and (**d**) final fast crack propagation region.

SEM images of fatigue fracture surface of a DP800/1.3-DP800/1.3 weldment tested under a maximum stress of 626 MPa are shown in Figure 12. Multiple crack initiation points were found, located at the surface, as shown in Figure 12b. Figure 12c illustrates the fatigue striations in the crack propagation region. The dimples in the final fast crack propagation region indicated the plastic fracture (Figure 12d).

**Figure 13:**
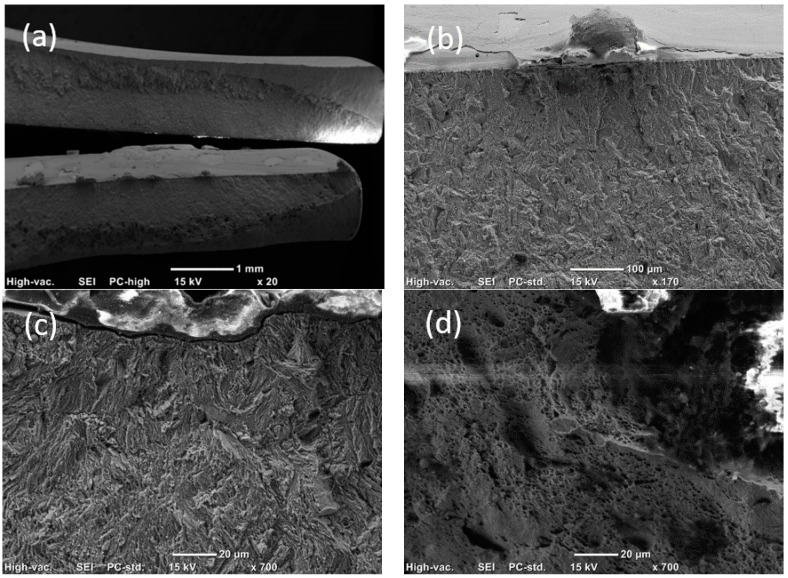
SEM images of a fatigue fracture surface of a DP1000/1.3-DP1000/1.3 weldment under stress 282 MPa. (**a**) Overall morphology of the fatigue fracture, (**b**) crack initiation region, (**c**) crack propagation region, and (**d**) final fast crack propagation region.

SEM images of a fatigue fracture surface of a DP1000/1.3-DP1000/1.3 weldment tested under the maximum stress level of 282 MPa are shown in Figure 13. Crack initiation from the surface of the weldment was observed. Impurity particles that appeared on the surface were also noticed. Fatigue striations were found in the crack propagation region, as shown Figure 13c. The obvious dimples at the fracture surface indicated the plastic fractures, meanwhile brittle fractures were noticed (Figure 13d).

## 4. Discussion

### 4.1. Microstructure Evolution

Different microstructural characteristics observed in the WJ could be a result from the different thermal cycles experienced during the welding processes due to the different absorption behavior of the different steel alloys and the differences in heat transport of the varying material geometries. The formation of large martensitic laths at the FZ is attributed to the large amount of heat and subsequent rapid cooling [13,18]. The peak temperature gradually decreases towards the BM, dividing the HAZ into HZ and SZ. As the HZ experienced thermal cycles with a peak temperature higher than Ac_3_, the ferrite and martensite in the BM were completely converted into austenite during the welding, and martensite is formed during the rapid cooling process. Furthermore, the decreasing peak temperature towards BM leads to the decreasing grain size in the HZ. The peak temperature decreases below the Ac_3_ with increasing distance from the weld center, leading to the formation of SZ. At the SZ close to the side of HZ, a fraction of ferrite and martensite is transformed into austenite, and the austenite part is converted into martensite and ferrite during the slow cooling cycle. As the temperature decreases further away from the FZ, the temperature will not reach Ac_1_, and tempered martensite is formed in the ferritic-martensitic structure.

### 4.2. Mechanical Properties

The fracture location and tensile test properties of the BM for DP800/1.3-DP800/1.3 and DP800/2.1-DP800/2.1 weldments indicate no significant effect of the WJ, which is ascribed to the narrow SZ and low degree of softening. A narrow SZ can be better restrained by the neighboring structure [28]. The optimized welding parameters, i.e., faster welding speed and lower laser power, could lead to a narrower softening zone and compress the effect of SZ on the strength [15,20]. Differently from DP800/1.3-DP800/1.3 and DP800/2.1-DP800/2.1 weldments, the yielding firstly occurred at the SZ for the DP1000/1.3-DP1000/1.3 weldment, due to the much lower microhardness values in the SZ when compared with the BM. The plastic deformation subsequently concentrates there, as failure was observed at the WJ. This, in turn, leads to reduction of the elongation. Therefore, the fracture occurring at the WJ and the tensile property in the DP800/2.1-DP1000/1.3 could be caused by the narrower soft zone and smaller extent of the SZ at the DP800 side while wider extention of the SZ was observed at DP1000 side. This could also explain the higher elongation and fracture location in the DP800/1.3-DP1000/1.3 weldment where the wider SZ at the DP1000 side could accommodate larger strain before failure compared to the narrower SZ in the DP800/2.1-DP1000/1.3 weldment.

The different locations of fracture after tensile and fatigue tests indicates that the narrow SZ does not affect the tensile properties, while the fatigue resistance was sensitive to the presence of the SZ. Although higher fatigue strength was reported in the DP steel with increasing martensite content [29,30,31], lower fatigue strength of DP1000/1.3-DP1000/1.3 weldment than DP800/1.3-DP800/1.3 weldment was found in this work, which could be a result of different surface defects as oxide particles appearing on the weld surface. In addition, the weld concavity can initiate formation of fatigue cracks [32,33]. The lower fatigue strength of DP800/2.1-DP800/2.1 weldment compared with DP800/1.3-DP800/1.3 weldment can be related to the presence of the weld concavity. All these findings confirm that not only the occurrence of SZ, but especially the defects introduced during welding process plays an important role on the fatigue fracture.

## 5. Conclusions

Microstructure of the welded joints and the effect of the welding on the tensile and fatigue properties for different combinations of similar/dissimilar materials with different thicknesses were investigated. Based on the results achieved, the following conclusions can be drawn:

The evolution trends of microhardness of the five types of the welded joints were consistent, featuring highest values in the fusion zone and lowest values in the soft zone. However, the degree of softening was more severe and the size of the SZ was wider for DP1000 steel than for the DP800 steel.

(1) A difference in thickness, (1.3 to 2.1 mm), for the welded DP800 steel, did not affect the tensile property significantly and the fracture occurred at the base material.
(2) The elongation at fracture of the DP1000/1.3-DP1000/1.3 weldment was greatly reduced in comparison with the tensile test values of the base material and the fracture occurred at the welded joint.
(3) The DP800/1.3-DP1000/1.3 weldment fractured at the base material and showed similar values of yield strength and ultimate tensile stress but lower elongation, compared to DP800/1.3-DP800/1.3 weldment. The DP800/2.1-DP1000/1.3 weldment fractured at the welded joint and showed the highest yield strength and ultimate tensile stress values in comparison with the other weldments, but it showed though the lowest elongation.
(4) The fatigue fractures occurred at the welded joint for all types of weldments. DP800/1.3-DP800/1.3 weldment had the highest fatigue value for 106 cycles, while DP800/2.1-DP1000/1.3 weldment showed the lowest. The fatigue fracture was initiated from the weld surface and the presence of concavities and impurities had a negative effect on the fatigue strength. The fatigue strength values of the thinner DP800 sheets (1.3 mm) showed larger scatter in comparison with the thicker sheets (2.1 mm).

## Figures and Tables

**Table 1:** Chemical compositions and mechanical properties of DP800 and DP1000.

Material	Chemical Composition (wt %)								Mechanical Properties		
	C	Mn + Al + Si	P	S	Cu	Ni	Cr + Mo	V + Nb + Ti	YS * (MPa)	UTS * (MPa)	EI * (%)
DP800	0.157	2.43	0.009	0.0020	-	-	-	0.0043	686	815	7.9
DP1000	0.113	2.68	0.014	0.0014	0.0123	0.174	0.038	0.0275	760	1041	8.0

* YS: yield strength; UTS: Ultimate tensile strength; EI: Elongation at fracture.

**Table 2:** Different welded joint (WJ) combinations.

Joint Identification	Base Metal A	Base Metal B
1#	DP800/1.3 mm	DP800/1.3 mm
2#	DP1000/1.3 mm	DP1000/1.3 mm
3#	DP1000/1.3 mm	DP800/1.3 mm
4#	DP800/2.1 mm	DP800/2.1 mm
5#	DP800/2.1 mm	DP1000/1.3 mm

**Table 3:** Welding conditions used in the present study.

Laser System	Focal Beam Diameter (µm)	Focal Distance (mm)	Laser Power (kW)	Welding Speed (m/min)
IPG fiber laser (YRL-15000)	660	250	3	3

**Table 4:** Tensile test results and fatigue limit at 10^6^ cycles.

Joint Identification	Yield Strength (MPa)	Ultimate Tensile Strength (MPa)	Elongation (%)	Fracture Location after Tensile test	Fatigue Limit (MPa)
1# (DP800/1.3)	701	868	7.9	BM	348
2# (DP1000/1.3)	883	1034	1.9	WJ	211
3# (DP800/1.3-DP1000/1.3)	747	858	5.1	BM	209
4# (DP800/2.1)	742	861	6.2	BM	221
5# (DP800/2.1-DP1000/1.3)	955	1075	1.2	WJ	<200

## Data Availability

The data presented in this study are available on request from the corresponding author.
